# Occlusal Plane Steepness and Profile Change Following TAD-Based One-Step Retraction on Four-Unit Extraction Cases: A Retrospective Study

**DOI:** 10.3390/diagnostics13142395

**Published:** 2023-07-18

**Authors:** Trefa Mohammed Ali Mahmood

**Affiliations:** Department of Pedodontics, Orthodontics and Preventive Dentistry, College of Dentistry, University of Sulaimani, Sulaimaniyah 46001, Iraq; trefa.ali@univsul.edu.iq

**Keywords:** steepness of occlusal plane, temporary anchorage devices, esthetics

## Abstract

Background: With the introduction of high-tech appliances, anchorage devices, and improved patient awareness of the risks associated with maxillofacial surgery, treating complex situations with orthodontic treatment has become more difficult in recent years. This study was conducted to demonstrate that orienting the occlusal plane, all the dental, skeletal, and soft tissue parameters, would be improved and to find which of these parameters could be correlated with the steepness of the occlusal plane. Materials and methods: This was a retrospective study including 40 cephalometric interpretations for patients who were planned for four-unit extractions (20 cephalometric radiographies before treatment and 20 after finishing the treatment). All were treated in the same orthodontic clinic with the same protocol using the McLaughlin–Bennett–Trevisi (MBT) prescription, with 22 slots and one-step retraction following four-unit extraction based on temporary anchorage devices (TADs). Results: There was no significant change in the canting of the occlusal plane, and it remained relatively stable from 6.31° to 7.55°, while all the soft tissue-related cephalometric measurements were reduced significantly, except the nasolabial angle, as the relation of the upper and lower lip to the esthetic line of Ricketts’ (E-Line) was reduced by 2.91 and 2.46°, respectively; furthermore, the angle of convexity was reduced from 10.92° to 9.79°. Besides, the upper incisor display was reduced by 0.38° Conclusions: Both the Frankfort mandibular angle and upper-incisor-to-Frankfort horizontal plane were significant parametric factors associated with profile change after extraction treatment having a positive 0.01-level Pearson association with occlusal plane steepness. Therefore, using the MBT prescription with TAD-based retraction is one of the favorable methods for the management of complex cases.

## 1. Introduction

The soft tissue appearance of the lower third of the face can be altered as a result of orthodontic treatment. In the nonextraction group, the lips and incisors moved forward, whereas they retreated in the extraction group in [1]. Alqahtani et al., 2020 [2], found that patients with bimaxillary protrusion who underwent extraction of the four second premolars and subsequent retraction of the anterior teeth exhibited profound soft tissue alterations. Contrary to the commonly held belief, orthodontists can also employ the extraction of the second premolars with a noticeable improvement in facial profile [2].

The debate about whether malocclusion can or should be treated with or without the extraction of the premolars continues. There is limited evidence that premolar extractions have either a negative or a positive effect on orthodontic treatment outcomes compared with the nonextraction treatment. The majority of studies had significant methodological weaknesses. Given these weaknesses, further systematic reviews in this area are highly unlikely to provide new information, and editors of academic orthodontic journals should discourage further reviews from being submitted and published. Investigators should concentrate on collecting primary data of the outcomes that are important to patients [3].

In addition to skeletal and dentoalveolar alterations, the goal of orthodontic therapy is to maintain or improve face aesthetics, particularly the soft tissue profile, which reflects changes brought on by the movements of the underlying hard tissue [4]. However, information on the relationships between standard cephalometric values and perceived facial beauty is still lacking [5].

Measuring the amount of incisor prominence can be difficult, particularly when considering changes over time in both lip and chin prominence [6]. Ramaut et al., 2019 [7], revealed that the perioral region of aging is impacted by a combination of volume loss and soft tissue lengthening and thinning, as the prediction of lip prominence based on hard tissue measurements could be useful in orthodontic treatment planning, but it has proven difficult and formidable thus far [8]. The two primary objective metrics associated with facial esthetics are lip protrusion and the position of the incisors beneath the lips. For facial esthetics, the position and contour of the lips are indispensable. The underlying upper and lower incisors to the Point A to pogonion (AP)-line and the upper and lower lips to the E-line have a significant negative effect on profile esthetics. Consequently, the retraction of the incisors and diminution of lip prominence significantly improves facial esthetics [9].

There are several fixed appliance procedures, and each of these techniques uses a different set of biomechanical principles to treat patients [10]. The goal is to improve lip function and dentofacial aesthetics as much as possible. Depending on the extent of necessary incisor retraction, clinical therapy varies [6].

According to earlier prospective research that evaluated soft tissue procumbency in extraction and nonextraction cases [11,12], study participants who had their maxillary and mandibular premolars extracted had straighter faces and upright incisors in both arches. In a similar vein, it has been claimed that the extraction of the first mandibular and maxillary premolars flattens the face shape by 2–3 mm in comparison to orthodontic treatment without extraction [13].

In the closure of an extraction space, it is necessary to generate both a force to move the teeth and a root-paralleling moment to move them. With a fixed appliance, there are two major ways to perform this by sliding the teeth along an archwire (sliding mechanics) or by tying the teeth tightly to archwire segments and moving the segments with a spring between them (closing loop mechanics) [6].

Each approach offers a number of noteworthy benefits and drawbacks. Sliding mechanics faces severe binding and friction resistance, which is a major drawback, but there is also the automatic creation of the root-paralleling moments at the extraction site (a big advantage). There is no frictional resistance in loop mechanics, which is a huge advantage, but it requires more time and complexity to tune the loop to produce a root-paralleling moment and keep it proportional to the force needed to close the space [14].

When faced with difficult instances such as the removal of the four first premolars with TADs and the subsequent prescription of MBT, no previous research has revealed the effect of the en masse retraction on the steepness of the occlusal plane and profile changes. Therefore, this study was conducted to determine which dental, skeletal, and soft tissue parameters would be improved by orienting the occlusal plane and to determine which of these factors could be associated with the steepness of the occlusal plane.

## 2. Materials and Methods

### 2.1. Study Registration

Ethical approval was obtained from the Ethics Committee at the College of Dentistry/University of Sulaimani, No. 38, on 3 May 2023.

### 2.2. Sample

From the database of a private orthodontic clinic, twenty patients (20–31 years old) were randomly selected to have forty cephalometric radiographs taken before and after treatment with four-unit extraction (maxillary and mandibular first premolars), planned to retract the anterior segment using maximum anchorage by temporary anchorage devices. Using a *t*-test power of 0.05 (a power of 0.8 and an alpha probability error of 0.05), the G power program, Version 3.1, was used to determine the necessary sample size. It was determined that a statistically significant difference in each of the metrics could be found with fewer than 15 samples. The sample size was increased to 20 patients to compensate for the low quality of the available cephalometric radiographs and written informed consent has been obtained from the patients. 

According to the Shapiro–Wilk test, some of the data were normally distributed, and others were not; this was taken into consideration in the statistical analysis, and all *p*-values were greater than 0.05.

### 2.3. The Study Protocol

This was a retrospective study using cephalometric radiographs that were taken before and at the end of orthodontic treatment (the duration of the treatment was between 20 and 22 months from July 2019 to November 2022). The exclusion criteria encompassed patients who had received functional appliance therapy or surgical orthodontic treatment or who exhibited congenitally absent teeth (excluding the third molars). All the radiographs were selected from the patients that were treated with the same protocol in which: following first premolar extraction, fixed orthodontic appliances of the MBT prescription with a 0.022-inch slot were bonded from the right 2nd molar to the left 2nd molar, and then, a 0.014-inch NiTi archwire was inserted and tied to each bracket with elastic ligatures. The arch wire sequences used were 0.012-inch NiTi, followed by 0.014-inch NiTi, 0.018-inch, or 0.016-inch NiTi (depending on the alignment of the teeth), 0.017- × 0.025-inch NiTi, and finally, 0.017 × 0.025 st.st. The leveling and alignment stage was considered complete when a 0.017 × 0.025 st.st. archwire could be passively placed. Once the leveling and alignment stage was complete, the retraction phase was initiated. Therefore, self-drilling temporary anchorage devices of 8–10 mm in length and 1.6 mm in diameter (SSEM, made in Korea) were inserted with a hand drill between the 2nd premolar and the 1st molar for both sides of the upper and lower arches as an anchorage for the retraction force. At this stage, the retraction phase was initiated with the use of maximum-anchorage TADs.

The radiographs were taken by the same dental radiologist as part of the patients’ regular orthodontic records, with the patients maintaining natural head positions (Bursone, 1967) with the teeth in occlusion and the lips relaxed [15].

### 2.4. Cephalometric Analysis

By combining skeletal, dental, and soft tissue parameters in a cephalometric analysis, one would be able to summarize the effect of extraction on the final treatment outcome.

**Figure 1:**
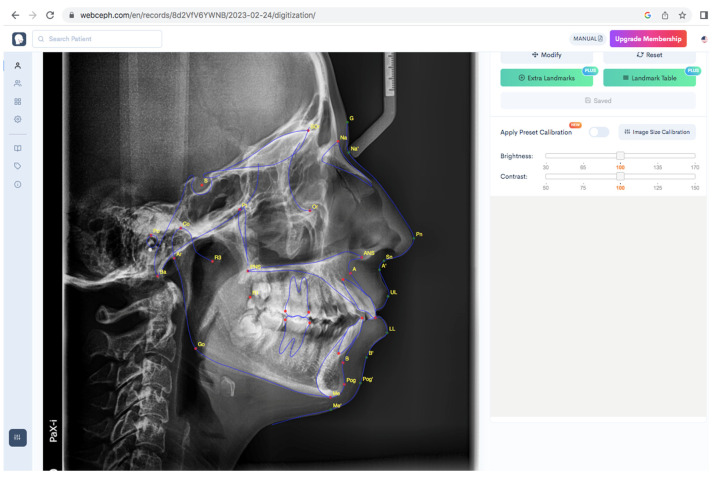
Cephalometric land mark used in the study. G: glabella, Na: nasion, Na’: soft tissue nasion, Pn: tip of the nose, Sn: subnasal, UL: upper lip anterior, LL: lower lip anterior, B’: soft tissue Point B, Pog’: soft tissue point pogonion, Me’: soft tissue menton, Or: orbital, S: midpoint of sella turcica, ANS: anterior nasal spine, PNS: posterior nasal spine, Pt: pterygomaxillary fissure, Go: gonion point, Pr: porion; Co: condylon; Ar: articulare; Ba; basion, A: Point A, B: Point B; R1: deepest point on the anterior border of the ramus; R3: deepest point on the sigmoid notch.

The Webceph software was used to perform the cephalometric analysis. By calibrating the real length of the ruler on the head positioner and simultaneously identifying the endpoints of the rulers and the anatomical landmarks, the magnifying likelihood was removed as in Figure 1. On the basis of previously published studies [16,17], the bone, dental, and soft tissue landmarks were determined.

**Figure 2:**
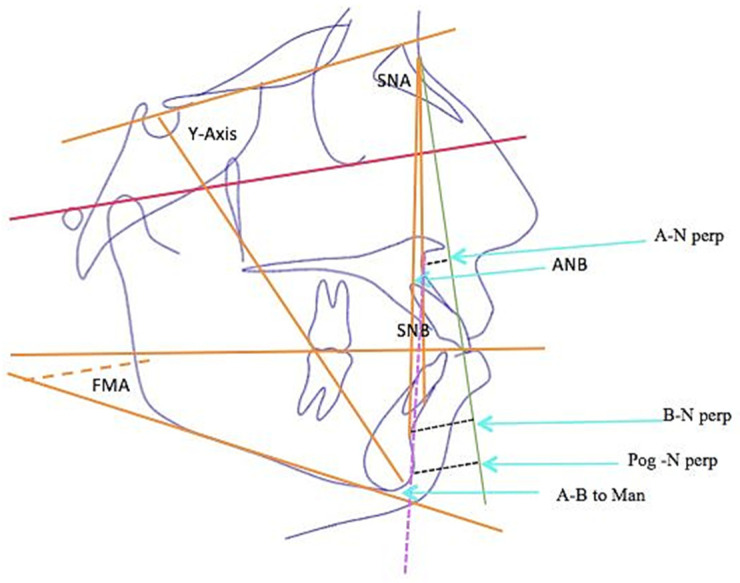
Skeletal parameters used in the study: SNA, SNB, and ANB represent the anteroposterior relation of the maxilla to the mandible; Y axis-FH and FMA represent the vertical relation; A-N prep, B-N perp, Pog-N perp, and A-B to Man. represent the position of the skeletal bases to the cranial base.

**Figure 3:**
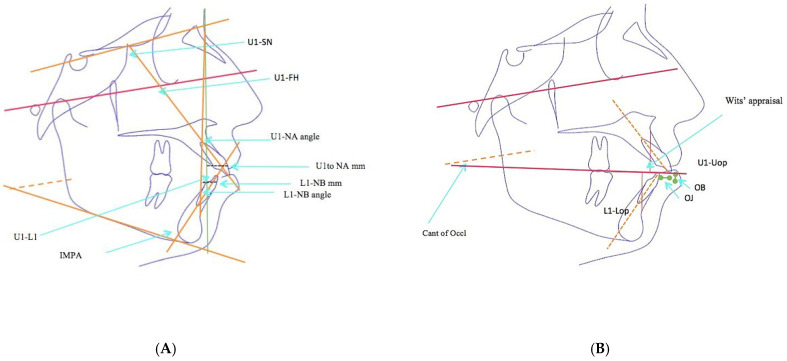
(**A**): Dental parameters used in the study: U1-SN, U1-FH, U1-NA angle, U1-NA mm, L1-NB angle, L1-NB mm, U1-L1 angle, IMPA. (**B**): Dental parameters used in the study: Wit’s appraisal, U1-Uop, OJ, OB, L1-Lop, Cant of Occl.

**Figure 4:**
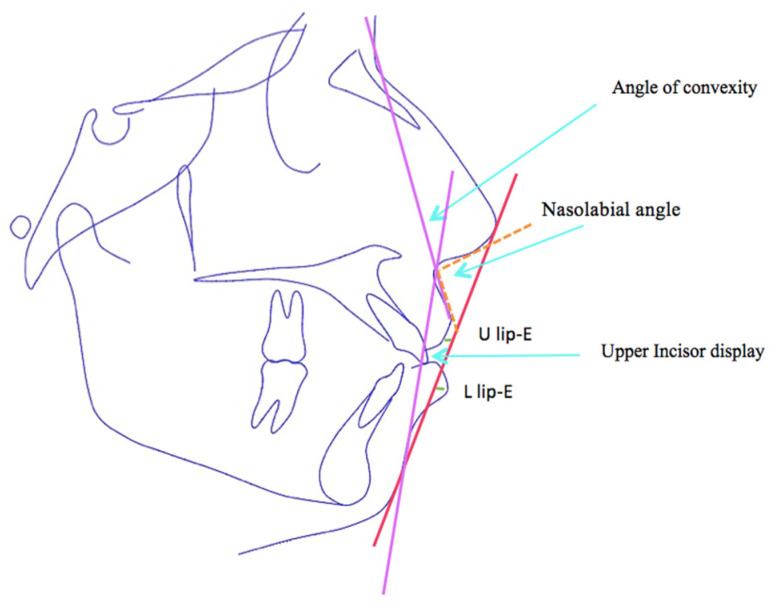
Soft tissue parameters used in the study: angle of convexity, nasolabial angle, U lip-E, L lip-E, upper incisor display.

In addition, the pretreatment and posttreatment cephalometric radiographs were superimposed on each other using the anterior cranial base anatomy, and the changes in each variable were quantified [18]. The manual identification of cephalometric landmarks on the digital pictures was performed by the same examiner. This was followed by the linear and angular variable measurements of the skeletal, dental, and soft tissue areas, using the appropriate analyses for each of these areas, and the same orthodontist managed all the cases with fixed MBT (0.022′′ slot) mechanotherapy using maximum-anchorage TADs in the upper and lower arches. On the basis of the reference planes and landmarks that were given, all of the linear and angular cephalometric measurements were recorded. Figure 2, Figure 3 and Figure 4 describe different skeletal, dental, and soft tissue profile measurements. A total of 28 measurements, including angular and linear measurements, were extracted for each individual before and after treatment, as shown in Table 1, Table 2 and Table 3.

### 2.5. Statistical Analysis

The same investigator traced and measured five of the randomly selected cephalometric radiographs in order to guarantee that there was consistency within the examination process. Both the locating of the cephalometric landmarks and the taking of the measurements of the variables took place in a total of two distinct sessions, which were spaced out by a time of two weeks from one another. The significance level was set at a *p* less than 0.05, and the range of the correlation values that were considered extremely dependable in order to evaluate the level of agreement showed that there was good agreement.

In order to determine whether or not there was an improvement in the profile, the correlation coefficients between the pre-cephalometric and post-cephalometric data were calculated and analyzed. It was determined how the en masse retraction would affect the steepness of the occlusal plane, taking into account difficult circumstances.

Version 25 of the Statistical Package for the Social Sciences was used to analyze the collected data. The variables of interest each had their own set of descriptive statistics computed for them. Using a paired *t*-test, the difference between the pretreatment and posttreatment cephalometric variables that were normally distributed was evaluated. In addition, Wilcoxon’s signed rank test was computed for all non-parametric variables. a *p*-value less than 0.05 was deemed statistically significant, and any *p*-value less than 0.01 was deemed extremely significant.

To find the correlation between the steepness of the occlusal plane and the significant variables, Spearman’s rank correlation was used for the normally distributed variables, and the Kruskal–Wallis test was used to find the correlation with the non-parametric variables. The significance level of the results was specified at a *p*-value of less than 5% (=0.05) and a 95% confidence interval.

## 3. Results

The values of A-B to Man, OB, L1 to Lop, interincisal angle, L lip to E-line, angle of Conv, and U incisor display increased significantly, while the values of SNA, SNB, Y-axis, Go angle, OJ, U1-FH, U1-SN, U1-Uop, IMPA, and U lip-E decreased significantly.

Both FMA and U1-FH were significant parametric factors associated with a profile change after extraction treatment having a positive, 0.01-level Pearson association with occlusal plane steepness.

**Figure 5:**
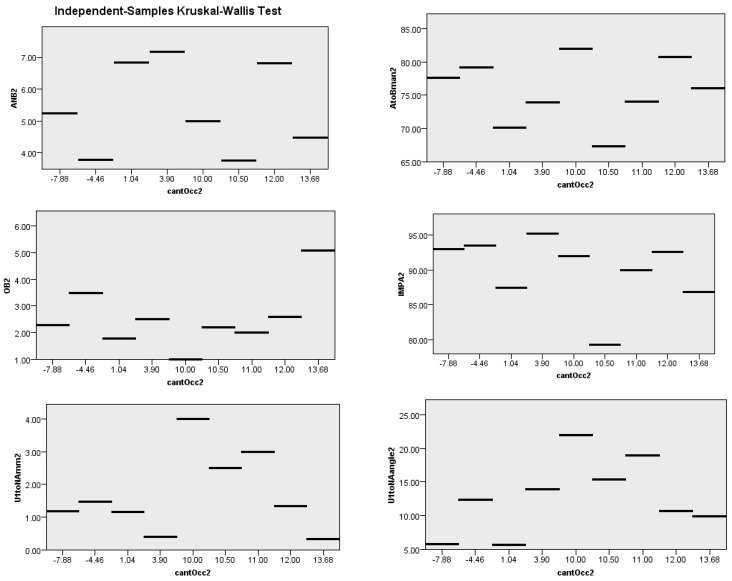

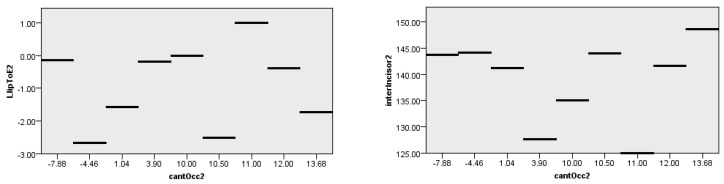
The distribution of the non-parametric variables using the Kruskal–Wallis test (ANB2, A-B-Man angle2, OB2, IMPA2, U1-NAmm2,U1-NA angle, interincisal angle, L lip-E2); the null hypothesis was rejected at a significant level > 0.05.

The Kruskal–Wallis test results for ABN, A-B-Man angle, OB, IMPA, U1-NAmm, U1-NA angle, interincisal angle, and lower lip-E-line are shown in Figure 5; these results showed that the null hypothesis was rejected at a level of significance greater than 0.05.

There was a reduction in the skeletal parameter of SNA and SNB by 1.36° and 0.53°, respectively, and only a 0.02° non-significant reduction of the angular value of ANB. However, there was a reduction in the value of the Y axis up to 4.83°, and the gonial angle showed a minimal amount of reduction of about 0.93°. On the other hand, there was an increase in the value of A-B to Man around 1.23 mm.

Considering the dental parameters, there was a reduction of about −1.32 for the amount of OJ and an increase in the amount of OB up to −1.32 mm. Furthermore, the issue related to upper incisor inclination, from different aspects such as the U1-FH, U1 to SN, U1-Uop, and U1-NA angle, revealed that there were reductions in these measurements by up to 16.08°, 18.03°, 9°, and 13°, respectively.

The interincisal angle was increased by −13.69° from 116.84° to 130.53°. On the other hand, the canting of the occlusal plane remained relatively stable from 6.31° to 7.55°.

The relation of the lower incisor changes was correlated with the L1 to Lop from 66.32° to 116.84°, as is clear from Table 2. The millimeter distance of the upper and lower incisors in relation to NA and AB was reduced significantly at a *p*-value of 0.000 from 5.70 to 1.66 mm and 9.14 to 5.03 mm, respectively.

From Table 3, all the soft-tissue-related cephalometric measurements were reduced significantly, except the nasolabial angle as the relation of U lip-E and L lip-E was reduced by 2.91 and 2.46°, respectively, and also, the angle of Conv was reduced from 10.92° to 9.79°. Besides, the upper incisor display was reduced by 0.38°.

**Figure 6:**
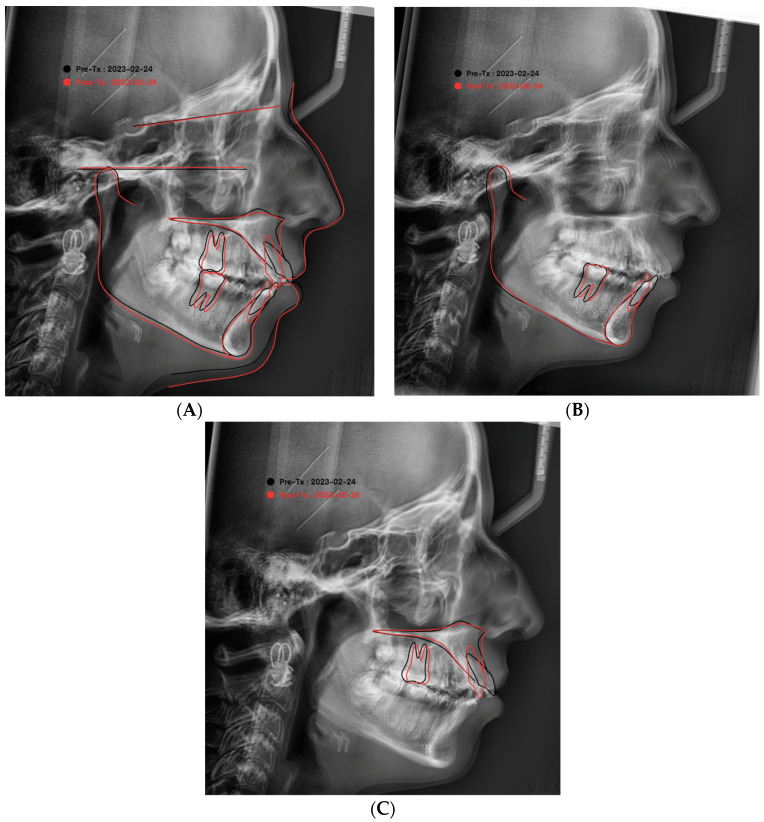
Super-imposition of the pretreatment and posttreatment tracing (**A**) over the SN plane, revealing the forward movement of the chin and retroclination of the upper incisors, (**B**) over the mandibular symphysis, showing the retroclination of the lower incisors (**C**), and over the Max plane, illustrating the retroclination of the upper incisors; overall, there was no loss of anchorage, which can be noticed by the position of the maxillary and mandibular molars.

Table 4 shows that there was a strong positive correlation between the steepness of the occlusal plane and FMA and a strong negative correlation with U1 to FH at a *p*-value of 0.000 and the super-imposition of the pretreatment and posttreatment tracing revealed the forward movement of the chin and retroclination of the upper incisors and the retroclination of the lower incisors; overall, there was no loss of anchorage as shown in Figure 6.

## 4. Discussion

Patients should not be treated without tooth extraction just to prevent tooth extraction or to make treatment more convenient, since this may jeopardize the outcome and stability of orthodontic treatment. Utilizing the appropriate extraction procedure for each type of malocclusion [11] is the optimal method. In other words, despite the growing popularity of nonextraction treatment, many orthodontic patients have inadequate space or congestion, necessitating extractions for a successful treatment outcome [15,19]. Although there is widespread consensus that orthodontic therapy can affect the facial profile, there is still some debate [20].

As a result, this research addressed the amplitude and reactivity of the soft tissue profile as a result of incisor placement modifications, as well as the influence of alternative premolar extraction sequences. Following a four-unit extraction in orthodontics, the degree to which the occlusal plane is steepened relies on a number of different circumstances. These considerations include the severity of the crowding, the degree of the extraction space, the position of the remaining teeth, and the treatment objectives. When four premolars are extracted, the occlusal plane has a tendency to grow flatter or even dip downwards toward the extraction site. This is because the remaining teeth fill in the space left by the premolars. This may be the result of the absence of the vertical support that was previously provided by the teeth that were extracted, as well as the intrusion of the teeth that were next to the extraction sites.

Orthodontists may employ a variety of procedures, such as differential anterior intrusion, differential posterior extrusion, or the placement of TADs, in order to keep the remaining teeth in their proper vertical position and prevent the occurrence of the aforementioned issue.

In addition, the ultimate placement of the occlusal plane should be determined not only by the desired outcomes of the orthodontic treatment, but also by the priorities of both the orthodontist and the patient. For patients who have a deep bite, for instance, the orthodontist may choose to purposefully flatten the occlusal plane in order to improve the patient’s bite, as well as the patient’s overall esthetics. On the other hand, if the patient has an occlusal plane that is flat, the orthodontist may attempt to produce an occlusal plane that is more curved in order to improve both the patient’s function and his/her appearance.

To summarize, the steepness of the occlusal plane following a four-unit extraction in orthodontics is a difficult issue that calls for careful planning and the consideration of numerous elements in order to obtain the outcomes that are sought from the therapy. Trisnawaty et al., 2013 [17], reported minimal retraction in the second premolar extraction. On the other hand, the results of the present research showed that there was a significant degree of upper incisor retraction. Additionally, a favorable correlation was found between the retraction of the upper incisors and the protrusion of the top and lower lips.

According to the findings of previous studies, the only circumstance in which the extraction of the first four premolars is considered appropriate is when a greater amount of lower incisor retraction would be desired as the ultimate result [21].

Surprisingly, the majority of studies have evaluated the perceived esthetics of frontal views rather than the actual profiles of people [22]. Therefore, a proper evaluation of facial angles and proportions is required to achieve posttreatment patient satisfaction with esthetic concerns [23], as Guimaraes et al. [24] concluded that treatment with dental extractions in patients with moderate protrusion generates impacts that can be more easily noticed in the profile analysis. However, in patients with excessive protrusion, changes promoted by treatment are noticed in frontal analysis in the same way as in profile. The improvements brought about by treatment are visible in frontal analysis, as well as in profile in individuals with severe protrusion. According to lay observers, the chin makes up the majority of facial attractiveness; however, according to orthodontists, the lips make up the majority. The contribution of teeth to facial attractiveness is substantially less than that of the lips and chin, whether in the opinion of laypeople or orthodontists [25].

Sadry et al., 2022 [26], implied that tooth extraction during orthodontic therapy may alter the thickness and axis of the vermilion upper lip, while the soft tissue facial profile is unaffected. To prevent unfavorable and detrimental consequences on the soft tissue profile of the face, premolar tooth extraction can be performed after a precise diagnostic and treatment plan has been established.

In maxillary protrusion patients enduring first premolar extraction, torque management of the maxillary incisors is essential. When retracting maxillary incisors in patients with bilateral maxillary first premolar extractions, Invisalign^®®^ was not as effective as Damon Q appliances. In patients undergoing bilateral maxillary first premolar extraction, Invisalign^®®^ increased the lingual inclination of maxillary incisors, notably in those with highly protruded maxillary incisors [27].

The McLaughlin–Bennet–Trevisi (MBT) prescription is a widely used orthodontic treatment approach that aims to achieve a harmonious and functional occlusion. The MBT prescription utilizes preadjusted brackets and wires to provide consistent and efficient tooth movement. In terms of the inclination of the occlusal plane, the MBT prescription does not dictate a specific inclination angle, but rather aims to achieve a level occlusal plane. However, the MBT prescription does provide guidelines for the angulation of individual teeth. Specifically, the brackets are designed to have a specific torque built into them, which is the degree of rotation or angulation of the bracket relative to the long axis of the tooth. This torque helps to control the inclination of the tooth in the sagittal plane and can influence the overall occlusal plane.

Additionally, the MBT prescription emphasizes the use of differential torque, where different teeth in the arch may have different degrees of torque applied to them to achieve optimal alignment and occlusion. This can help to achieve a level occlusal plane and maintain proper occlusal relationships between the upper and lower teeth.

In summary, while the MBT prescription does not dictate a specific inclination angle for the occlusal plane, it does provide guidelines for the angulation of individual teeth and emphasizes the use of differential torques to achieve optimal alignment and occlusion.

The alterations in the patient’s dentofacial structures can be better understood by superimposing cephalometric tracings taken before and after therapy. To visually compare the two images and evaluate the magnitude and direction of the alterations, we can overlay the pretreatment cephalometric tracing on the posttreatment tracing, using a shared reference point or landmark.

Several techniques exist for comparing pretreatment and posttreatment cephalometric tracings. The sella turcica, a bony feature in the middle of the skull, is often used as a reference point. The sella turcica is superimposed on both pictures to align the pretreatment and posttreatment tracings. This provides a means of contrasting the evolution of cranial and dental architecture in relation to the location of the sella turcica. The success of treatment, the durability of the results, and the necessity for further treatment can all be assessed by superimposing cephalometric tracings taken before and after. It can also be used to spot places where adjustments are needed for the best outcomes.

Compared to conventional 2D imaging methods such as cephalometric radiography, cone beam computed tomography (CBCT), imaging subjects the patient to a higher level of radiation exposure. While a single CBCT scan only exposes one to a small quantity of radiation, repeated scans over time can increase one’s chance of developing radiation-related health problems. As a result, the use of CBCT needs to be justified and compared to the advantages and disadvantages for each patient.

Additionally, CBCT imaging is more expensive and time-consuming than conventional 2D imaging techniques, which may make it unnecessary for ordinary orthodontic situations and raise the cost of treatment for patients. Cephalometric radiography is a suitable and appropriate imaging tool for treatment planning and evaluation in many orthodontic situations.

It is crucial to understand that changes in the soft tissues of the face alter more dramatically than changes in the jaw and the face’s hard tissues with growth. For orthodontists, the most-significant alteration is the downward sagging of the lips and other soft tissues of the face with age. As a result, the lower incisors are exposed more, while the upper incisors are exposed less [6]. A man’s maxilla and mandible move forward from the ages of 37 to 77, while a woman’s jaws expand forward and somewhat downward between the ages of 34 and 83 [6].

In a study published in 2021 by Knigge et al., the facial type influences the skeletal bases’ growth directions, causing the maxilla to rotate slightly anteriorly and downward in the hyperdivergent form, and the maxilla also grows slightly shorter. The condyle and coronoid process move anteriorly as the mandibular ramus becomes more vertically oriented with maturity, whereas the anterior maxilla rotates somewhat downward in both the normodivergent and hypodivergent forms. With the exception of the related rearward rotation of the corpus in the mandibular ramus, the normodivergent type exhibits growth that is comparable to growth in the hyperdivergent type. Despite being comparable to the normodivergent corpus, the mandibular symphysis grows much longer with age and projects more anteriorly while retaining its relative height [28].

Premolar extractions have a far smaller impact on facial profile than is commonly believed, with most patients noticing only a modest shift in lip profile. Concerns regarding the worsening of the facial profile and the loss of vertical dimension should not be the primary basis for the extraction choice in integrated treatment planning. Whether or not to extract healthy premolars in patients on the cusp of needing extraction therapy is a challenging issue. This research elucidated the consequences of premolar extraction on cephalometric dentofacial characteristics, paving the way for more-informed orthodontic extraction choices in the future [29].

The limitation of this study could be attributed to lacking information about smile analysis (such as: smile arc, buccal corridors, tooth size, shade, etc.) as the evaluation was performed only by cephalometric analysis, revealing the profile and vertical relation. Besides, long-term follow up is essential to qualify the effect of age on the treatment outcome.

## 5. Conclusions

Both the Frankfort mandibular angle and upper incisor to Frankfort horizontal plane were significant parametric factors associated with profile change after extraction treatment, having a positive, 0.01-level Pearson association with occlusal plane steepness. Therefore, using the MBT perception with TAD-based retraction is one of the favorable methods for the management of complex cases. Concerns regarding the worsening of the facial profile and a loss of the vertical dimension should not be the primary basis for the extraction choice in integrated treatment planning.

## Figures and Tables

**Table 1:** Descriptive statistics, mean difference between pretreatment and posttreatment values, and paired *t*-test significance among the skeletal-related cephalometric measurements.

Skeletal Parameters		Normal Values	No.	Results		Mean Diff.	Sig. (2-Tailed)	Wilcoxon Related Test
		Mean ± Sd		Mean	Sd			
1.	SNA	81.08 ± 3.7	20	81.88°	4.05	1.36	0.000 *	
	SNA2		20	80.52°	3.64			
2.	SNB	79.17 ± 3.8	20	76.23°	3.55	0.53		0.001 *
	SNB2		20	74.70°	2.72			
3.	ANB	2.46 ± 1.8	20	5.47°	1.58	0.02	0.863	
	ANB2		18	5.45°	1.30			
4.	Y axis-FH	59 ± 6	20	61.95°	4.78	4.83		0.014 *
	Y axis-FH 2		20	57.12°	11.16			
5.	FMA	25 ± 4	20	25.7°	6.14	2.1	0.000 *	
	FMA2		20	23.58°	6.51			
6.	A-N perp	0.4 ± 2.3	20	2.3	5.62	0.23		0.654
	A-N perp2		20	2.07	5.41			
7.	B-N prep	−3.5 ± 2	20	−4.49	8.99	−10.29		0.167
	B-N perp2		20	−5.8	8.36			
8.	Pog-N perp	−1.8 ± 4.5	20	−4.12	10.03	1.5		0.231
	Pog-N perp2		20	−5.62	9.10			
9.	A-B to Man1	69.3 ± 2.5	20	74.71	5.71	−1.31	0.002 *	
	A-B to Man2		20	76.02	4.62			

Sd = standard deviation, mean diff. = mean difference,* sig. = significant.

**Table 2:** Descriptive statistics, mean difference between pretreatment and posttreatment values, and paired *t*-test significance among the dental-related cephalometric measurements.

Dental Variables		Normal Values	No.	Results		Mean Diff.	Sig. (2-Tailed)	Wilcoxon Related Test
		Mean ± Sd		Mean	Sd			
1.	Wit’s appraisal	−2.74 ± 0.3	20	3.05	1.69	0.65	0.057	
	Wit’s appraisal2		20	2.40	2.46			
2.	OJ	2 ± 2	20	4.01	1.73	1.23	0.002 *	
	OJ2		20	2.78	1.21			
3.	OB	2 ± 2	20	1.22	2.59	−1.32		0.038 *
	OB2		20	2.54	1.07			
4.	U1-FH	113.8 ± 6.4	20	118.82°	4.75	16.08	0.000 *	
	U1-FH2		20	102.74°	5.42			
5.	U1-SN	105.28 ± 6.6	20	109.88°	5.94	18.03	0.000 *	
	U1-SN2		20	91.85°	5.88			
6.	U1-Uop2	55 ± 4	20	54.39°	3.92	9	0.000 *	
	U1-Uop		20	63.37°	6.83			
7.	IMPA	90 ± 3.5	20	104.02°	7.37	13.76		0.000 *
	IMPA2		20	90.26°	4.57			
8.	L1-Lop	66 ± 5	20	55.8	5	−10.51	0.000	
	L1 to Lop		20	55.81°	4.90			
	L1-Lop2		20	66.32°	4.87			
9.	U1-L1	130 ± 5.8	20	116.84°	13.62	−13.69		0.000 *
	Interincisal angle2		20	130.53°	15.67			
10.	Cant of Occl	9.3 ± 3.8	20	6.31°	4.63	−1.24		0.282
	Cant of Occl2		20	7.55°	6.03			
11.	U1-NA mm	4 ± 3	20	5.70	2.36	4.04		0.000 *
	U1-NA mm2		20	1.66	1.13			
12.	U1-NA angle	22 ± 5	20	25.29°	6.57	13		0.000 *
	U1-NA angle2		20	12.29°	5.33			
13.	L1-NB mm	4 ± 2	20	9.14	1.70	4.11	0.000 *	
	L1-NB mm2		20	5.03	1.43			
14.	L1-NB angle	25 ± 5	20	36.2°	4.72	13.41	0.000 *	
	L1-NB angle2		20	22.79°	4.39			

Sd = standard deviation, mean diff. = mean difference, * sig. = significant.

**Table 3:** Descriptive statistics, mean difference between pretreatment and posttreatment values, and paired *t*-test significance among the soft tissue-related cephalometric measurements.

Soft Tissue Variables		Normal Values	No.	Results		Mean Diff.	Sig. (2-Tailed)	Wilcoxon Related Test
		Mean ± Sd		Mean	Sd			
1.	U lip to E	−4.7 ± 2	20	−1.12	1.41	2.91	0.000 *	
	U lip to E2		20	−3.65	1.03			
2.	L lip to E	−2 ± 2	20	1.61	1.55	2.46		0.000 *
	L lip to E2		20	−0.85	1.17			
3.	Nasolabial angle	95 ± 5	20	94.85°	9.66	−1.6		0.55
	Nasolabial angle 2		20	96.45°	6.96			
4.	Angle of Conv	0 ± 5.1	20	10.92°	3.54	1.13	0.02 *	
	Angle of Conv2		20	9.79°	3.24			
5.	U incisor display	2.5 ± 1.5	20	3.18	2.43	0.38		0.145
	U incisor disply2		20	2.80	1.13			

Sd = standard deviation, mean diff. = mean difference, * sig. = significant.

**Table 4:** Spearman rank correlation between the steepness of occlusal plane and the significant parametric variables related to the profile change postextraction treatment.

Spearman’s Rank Cant Occlusal	*N*	Correlation Coefficient	Sig. (2-Tailed)
SNA2	20	−0.142	0.550
FMA2	20	0.717 **	0.000
Gon angle2	20	0.322	0.167
OJ2	20	309	0.185
U1 to FH2	20	−0.894 **	0.000
U1 to SN2	20	0.007	0.977
U1 to Uop2	20	−0.325	0.162
L1 to Lop2	20	0.422	0.064
L1 to NBmm2	20	−0.130	0.585
L1 to NBangle2	20	−0.072	0.761
Angle convex2	20	0.107	0.653

** Correlation is significant (Sig.) at the 0.01 level (2-tailed).

## Data Availability

Data are available from the corresponding author upon request.

## Abbreviations

Abbreviation	Description
AP-line	The angle between long axis of the most-anteriorly positioned maxillary incisor and the line passing through Point A and the pogonion
SNA	The angle between SN-line and NA-line: sagittal position of maxilla
SNB	The angle between SN-line and NB-line: sagittal position of mandible
ANB	The angle between Point A, nasion, and Point B: sagittal base relation of jaws
FMA	The angle between the mandibular plane and Frankfort horizontal plane
Y axis-FH	The angle formed by the intersection between the sella gnathion and the FH plane estimate of the mandibular growth direction; also indicates the degree of the downward and forward position of the chin in relation to the upper face
A to N perp (FH)	The perpendicular distance from Point A to A-N when it is drawn perpendicular to the FH plane
B to N perp (FH)	The perpendicular distance from Point B to B-N when it is drawn perpendicular to the FH plane
Pog to N perp (FH)	The perpendicular distance from Pog to Pog-N when it is drawn perpendicular to the FH plane
FH to AB	The angle between the line connecting Point A to Point B and the Frankfort horizontal plane
A-B to mandibular plane	The angle between the line connecting Point A to Point B and the mandibular plane
Wit’s appraisal	The millimeter distance between two perpendicular lines, from Point A and Point B onto the occlusal plane.
OJ	The vertical distance between the incisal ridges of the most-anteriorly positioned maxillary and mandibular incisors
OB	The sagittal distance between the incisal ridge of the most-anteriorly positioned maxillary incisor and the labial surface of the most-anteriorly positioned mandibular incisor
U1 to FH	The angle between the long axis of the most-anteriorly positioned maxillary incisor and the Frankfort plane
U1 to SN	The angle between the long axis of the most-anteriorly positioned maxillary incisor and SN plane (inclination of upper incisors)
U1 to Uop	The angle between the upper inclination line and the upper occlusal line
IMPA	The angle formed by the long axis of the most-anteriorly positioned lower incisor and the mandibular plane (lower incisor inclination)
L1 to Lop	The angle between the lower incisor inclination line and the lower occlusal line
Interincisal angle	The interincisal angle between the long axes of the most anteriorly positioned maxillary and mandibular incisors
Cant of occlusal plane	The steepness of the occlusal plane in relation to the mandibular plane
U1 to NA (mm)	The perpendicular distance from the most-prominent point on the labial surface of the upper incisor to the NA-line
U1 to NA (deg)	The angle formed by the upper inclination line and the NA-line
L1 to NB (mm)	The perpendicular distance from the most-prominent point on the labial surface of the lower incisor to the NB-line
L1 to NB (deg)	The angle formed by the lower inclination line and the NB-line
Upper lip to E-plane	The distance of the lower lip to the aesthetic line (E-line), perpendicular to the E-line
Lower lip to E-plane	The distance of the upper lip to the aesthetic line (E-line), perpendicular to the E-line
Nasolabial angle	The angle formed by the two lines passing through the lower edge of the nose (the columella) and the edge of the upper lip
Upper incisal display	The display of the upper incisor on the lateral cephalogram
